# Serum Levels of Zinc, Albumin, Interleukin-6 and CRP in Patients with Unipolar and Bipolar Depression: Cross Sectional Study

**DOI:** 10.3390/cimb46050275

**Published:** 2024-05-09

**Authors:** Tihana Bagarić, Alma Mihaljević-Peleš, Milena Skočić Hanžek, Maja Živković, Ana Kozmar, Dunja Rogić

**Affiliations:** 1 Department for Psychiatry and Psychological Medicine, University Hospital Centre Zagreb, 10000 Zagreb, Croatia; apeles@mef.hr (A.M.-P.); mskocic@kbc-zagreb.hr (M.S.H.); maja.zivkovic@kbc-zagreb.hr (M.Ž.); akozmar@kbc-zagreb.hr (A.K.); dunjarogic@hotmail.com (D.R.); 2 School of Medicine, University of Zagreb, 10000 Zagreb, Croatia; 3 Faculty of Pharmacy and Biochemistry, University of Zagreb, 10000 Zagreb, Croatia

**Keywords:** bipolar depression, unipolar depression, zinc, albumin, CRP, IL-6, inflammation

## Abstract

Unipolar (UD) and bipolar depression (BDD) show a high degree of similarity in clinical presentations, which complicates the differential diagnosis of these disorders. The aim of this study was to investigate the serum levels of interleukin 6 (IL-6), C-reactive protein (CRP), albumin (Alb), and zinc (Zn) in patients with UD, BDD, and healthy controls (HC). A total of 211 samples were collected: 131 patient samples (65 UD and 68 BDD) and 80 HC. The Montgomery–Asberg Depression Rating Scale (MADRS), along with the Hamilton Depression Rating Scale (HAMD-17), were administered to patient groups to evaluate symptoms. A cross-sectional study was performed to analyse the serum levels of IL-6, CRP, albumin, and zinc. The concentration of CRP was determined using the immunoturbidimetry method, zinc using the colorimetric method, and albumin using the colorimetric method with bromocresol green on the Alinity c device. IL-6 cytokine concentration in serum samples was ascertained using a commercial enzyme immunoassay, ELISA. We found no significant differences in serum concentrations of zinc, albumin, CRP, and IL-6 between the groups of patients with unipolar and bipolar depression. There was a significant statistical difference (*p* < 0.001) between serum levels of all investigated parameters in both groups of depressed patients in comparison with HC. Furthermore, correlations with specific items on HAMD-17; (namely, hypochondrias, work and activities, somatic symptoms-general, and weight loss) and on MADRS (concentration difficulties, lassitude) were observed in both patient groups. These findings confirm the presence of low-grade inflammation in depression, thus adding better insight into the inflammation hypothesis directed to explain the aetiology of depressive disorders. Our results do not indicate potential biomarkers for distinguishing between unipolar and bipolar depression.

## 1. Introduction

At least 20% of the general population, at some point in their lives, experience an episode of mood swings, or even develop a mood disorder. Within mood disorders, bipolar disorder (BD) and depressive disorder (DD) are the most common and most disabling disorders [1,2]. Among patients who clinically present with depression, there is a significant number of those who later turn out to have bipolar disorder, that is, who belong on the bipolar spectrum [3,4,5]. Namely, a depressive episode in unipolar depression and a depressive episode in BD are clinically presented almost identically. Without insight into the longitudinal course of the disorder, it is difficult to distinguish which disorder it is at first. Even then, it should be taken into account that the onset of BD is characterized by alternations of depressive episodes (before the development of a hypomanic or manic episode) that can last for several years [6]. Furthermore, BD is clinically demanding to diagnose due to subsyndromal states that often occur between major episodes of the disorder [7].

Timely diagnostic differentiation of these disorders is important because pharmacotherapeutic treatment is fundamentally different. While unipolar depression is primarily treated with antidepressants, they are not a good therapeutic choice for bipolar disorder. Treatment with antidepressants in undiagnosed bipolar patients causes mixed states, faster exchanges of polarity, but also increases the risk of suicide, especially in younger age groups of patients [8,9].

Apart from anamnestic and hetero-anamnestic data and a clinical examination, for now, there is no objective measurable indicator that would help in the differential diagnosis of these two disorders.

The prevailing paradigm is that unipolar and bipolar depression are qualitatively and etiologically different [10]. This duality is also represented in the DSM 5 diagnostic manual, which categorizes them as separate disorders [11].

In recent years, numerous studies have suggested the involvement of the immune system and inflammatory processes in mood disorders [12,13,14].

Research indicates that both unipolar depression (UD) and bipolar disorder (BD) are associated with an inflammatory state characterized by imbalances in proinflammatory and anti-inflammatory cytokines [15,16]. While some studies report no significant differences in inflammatory markers like IL-6 and TNFR between UD and BD, other research highlights distinct immunological profiles for each disorder [13,17]. Specifically, patients with UD have shown elevated levels of cytokines such as IL-1β, TNF-α, and IL-12, whereas those with BD exhibit higher levels of IL-6, IL-18, IL-33, and sST2. These findings suggest differential immune-inflammatory involvement in the pathogenesis of UD and BD, although the data remain somewhat inconsistent across studies.

Biomarkers such as lL-6 [18,19,20,21], CRP [22,23], albumin [24,25], and zinc [26,27,28,29] have been subject of numerous studies aimed to shed light into complex interplay between inflammatory process and depressive symptoms. However, the differences in these biomarkers have not previously been evaluated in patients with UD and BD, and the relationship between these biomarkers and symptoms in BD and UD had not yet been studied.

Our study was designed to include a wider range of biological parameters in groups of hospitalized patients with BDD and hospitalized patients with UD and HC.

Four biological parameters were selected that in earlier research were found to be associated with inflammatory events in mood disorders: IL-6, CRP, Zn, and albumin levels [30,31].

The hypothesis was that, respecting the paradigm of the etiological diversity of these two disorders [32], there would be a significant difference in the concentration of the mentioned parameters between all examined groups.

We additionally considered, if one of the researched parameters would prove to be a good and clinically relevant biological marker for the distinction of these two disorders, it would be of great importance in daily clinical work and a great help when establishing a diagnosis.

## 2. Materials and Methods

### 2.1. Participants and Procedures

A total of 211 participants of both genders aged 18 to 60 were included in this cross-sectional study conducted in the period between 2016 and 2023: 65 patients with unipolar depression (UD), 68 patients with bipolar depression (BDD) treated in the Psychiatric Hospital “Dr. Ivan Barbot” in Popovača and in the Clinic for Psychiatry and Psychological Medicine, UHC Zagreb, and 80 healthy controls (HC). The diagnosis of UD and BDD was confirmed using a structured clinical interview based on the DSM 5 criteria [11]. All patients were experiencing an acute depressive episode at the time of admission to the hospital. We chose a consecutive sample of patients by the order of their arrival at the hospital treatment. The purpose of the research was explained to all participants who were suitable for participation in the study, and their informed consent was obtained. To assess the severity of depressive symptoms among patients, we used the Montgomery–Asberg Depression Rating Scale (MADRS) [33] and the Hamilton Rating Scale for Depression (HAMD-17) [34]. The study included patients whose depressive episode was evaluated at least as moderately severe using the clinical scales used (MADRS > 20, HAMD-17 > 18). The exclusion criteria were: refusal to give informed consent or withdrawal of informed consent to participate in research, clear manifestation of infection, physical diagnoses such as autoimmune diseases or malignant diseases, other psychiatric disorders or intellectual disability, and pregnancy and lactation. Also, we excluded participants on an extreme diet, those with an eating disorder, and those who abused psychoactive substances. The use of regular pharmacotherapy (according to the protocol for UD and BD) that patients used before the onset of the depressive episode was not in the exclusion criteria. As a control group, 80 healthy individuals matched by age and gender were recruited. The control group participants had no history of psychiatric disorders, and participation was voluntary in all cases. The same exclusion criteria were used for both the experimental and control groups. The study protocol was approved by the Ethics Committees of Psychiatric Hospital “Dr. Ivan Barbot” Popovača, University Hospital Centre Zagreb and Medical School of the University of Zagreb. The study complied with World Medical Association Declaration of Helsinki 2013 (World Medical Association 2013).

### 2.2. Collection of Blood Samples

From the patients diagnosed with UD and BDD blood samples were taken for the analysis of serum levels of interleukin 6 (IL-6), C-reactive protein (CRP), albumin (Alb), and zinc (Zn). Blood samples were taken from the cubital vein at around 8 am in tubes with a gel separator of 8 mL (Greiner Bio-One International GmbH, Kremsmünster, Austria).

The samples were then centrifuged for 10 min at 3000 rpm (Hettich Rotofix 32, Andreas Hetich Gmb & Co. KG, Tuttlingen, Germany). The obtained serum was divided into plastic tubes (1.5 mL; Kartell, Noviglio, Italy) which were stored at −20 °C for determination of interleukin 6 (IL-6). The serum sample for C-reactive protein (CRP), zinc, and albumin tests were analysed using centrifugation.

### 2.3. Statistical Analysis

Categorical data are represented by absolute and relative frequencies. Differences in categorical variables were tested with the Chi-square test and, if necessary, with Fisher’s exact test. The normality of the distribution of numerical variables was tested with the Shapiro–Wilk test. Numerical data are described by the median and the limits of the interquartile range. Differences of numerical variables between two independent groups was performed using the Mann–Whitney U test (with the Hodges–Lehmann median difference and 95% confidence interval of the difference shown). The internal reliability of the scales (HAM-D and MADRS) was expressed through the Cronbach Alpha coefficient. The association of continuous variables was assessed using Spearman’s correlation coefficient ρ (rho). For all multiple testing, the Bonferroni correction was used. The influence of independent factors on the severity of a depressive episode and changes in depressive illness were tested using bivariate and multivariate logistic regression (without correction and with correction for taking antidepressants). All *p* values are two-sided. The significance level was set at alpha (α) = 0.05. The statistical program MedCalc^®^ Statistical Software version 22.006 (MedCalc^®^ Statistical Software version 22.018 (MedCalc Software Ltd., Ostend, Belgium); https://www.medcalc.org (accessed on 6 May 2024)) and SPSS (ver.23.0, SPSS Inc., Chicago, IL, USA) were used for statistical analysis.

### 2.4. Determination of CRP, Zinc, and Albumin

The concentration of CRP was determined using the immunoturbidimetry method, zinc using the colorimetric method, and albumin using the bromcresol green colorimetric method on the Alinity c device (Abbott Laboratories, Chicago, IL, USA). All methods for CRP and albumin were implemented and validated according to [35]. 

### 2.5. Determination of IL-6

IL-6 cytokine concentration in serum samples was determined using a commercial enzyme immunoassay, ELISA (Enzyme-linked immunosorbent assay). Assays were performed according to the manufacturer’s instructions (Invitrogen, Thermo Fisher Scientific Inc., Waltham, MA, USA).

### 2.6. Performing an ELISA Test

The wells of the microtiter plate coated with monoclonal antibody to human IL-6 were washed twice with 300 µL of washing solution each. After washing, add 100 µL of the standard (concentration range 5.0–0.05 pg/mL) to the wells and 50 µL of the serum diluent and 50 µL of the sample to the serum wells. Then, 50 µL biotin-labelled monoclonal anti-human IL-6 antibody was added to all wells and incubated at room temperature for two hours on a Mini-Shaker PSU-2T mixer (BioSan, Riga, Latvia). After incubation, the plate was washed six times, and 100 µL of freshly prepared streptavidin-HRP conjugate was added to the wells and incubated at room temperature for one hour on a mixer. After washing again, add 100 µL of amplification solution I to all wells and incubate for 15 min at room temperature on a mixer. Washing the plate again precedes adding 100 µL of amplification solution II to all wells and incubation for 30 min on a mixer. After the final washing, 100 µL of tetramethyl benzidine (TBM) substrate was added to the wells and incubated for 20 min protected from light. The reaction was stopped by adding 100 µL of 1 M phosphoric acid. The absorbance of the resulting developed colour is immediately read at 450 nm with a reference wavelength of 620 nm on a Sunrise microtiter plate reader (Tecan Trading AG, Männedorf, Switzerland). The measurement results were calculated according to the standard curve (software support Magellan 7.3 STD Tecan Trading AG, Männedorf, Switzerland) and multiplied using the appropriate dilution factor and expressed in pg/mL. The standard curve was obtained from standards of known concentrations.

## 3. Results

### 3.1. Sociodemographic and General Medical Characteristics of Study Participants

This research was conducted on 211 participants, of whom 131 (62.1%) were patients with depression. In total, 66 subjects (50.4%) had bipolar and 65 subjects (49.6%) had unipolar depression. The healthy control group consisted of 80 participants. We found no significant difference in relation to the gender and mean age between the control group and the patient group (Table 1).

In total, 84 (64%) participants are women and 47 (36%) are men, with no significant difference in relation to the spectrum.

The most common diagnosis is bipolar depression—moderately severe depressive episode in 60 (46%) subjects, and depressive disorder—moderately severe depressive episode in 44 (34%) subjects.

Median age of the participants is 55 (interquartile range from 47 to 58 years) in the range from 52 to 60 years, without a significant difference in relation to the spectrum. In total, 103 (79%) respondents have children, with no significant difference in the number of children compared to the examined groups. In total, 75 (56%) participants have a high school education, and 17 (13%) have a university degree, of which there are significantly more, 16 (24%) of them from the group of participants with bipolar depression (χ^2^ test, *p* = 0.004). In total, 59 (45%) participants are employed, and 40 (31%) are retired. In total, 78 (60%) participants are married, 24 (18%) are single, 22 (17%) participants are divorced, and 7 (5%) are widowed. Again, no statistically significant difference between examined groups was found in all of the above listed parameters.

Psychiatric heredity was noted in 42 (32%) participants.

Interestingly, in the group of subjects with bipolar depression who smoked tobacco cigarettes, we found a significantly higher number of packs of cigarettes per day (Mann–Whitney U test, *p* = 0.023) and “pack years” (Mann–Whitney U test, *p* = 0.018) compared to group of study participants who have unipolar depression (Table 2).

### 3.2. Serum Levels of Il-6, CRP, Alb and Zn in Patients and Healthy Control Subjects

The values of IL-6 (Mann–Whitney U test, *p* = 0.006), CRP (Mann–Whitney U test, *p* = 0.002), and zinc (Mann–Whitney U test, *p* = 0.013) were significantly higher in the patient group compared to the control group, while albumin values were significantly lower in the patient group compared to the control (Mann–Whitney U test, *p* = 0.002) (Table 3).

### 3.3. Comparison of Serum Levels of Il-6, CRP, Alb, and Zn in Patients with Depressive Episode of Bipolar Disorder (DBD), Unipolar Depression (UD), and Healthy Control Group (HC)

There are significantly lower values in the control group compared to patients with unipolar or bipolar spectrum in levels of IL-6 (Kruskal–Wallis test, *p* = 0.020) and CRP (Kruskal–Wallis test, *p* = 0.008), while albumin values are significantly higher (Kruskal–Wallis test, *p* = 0.007). Zinc values are significantly lower in the control group compared to the group of patients with unipolar spectrum (Kruskal–Wallis test, *p* = 0.013), while they do not significantly differ from the values of patients with bipolar spectrum (Table 4).

### 3.4. Comparison of Serum Levels of Il-6, CRP, Albumin, and Zinc in BD and UD Subjects

There are no significant differences in the values of zinc, albumin, CRP, and IL-6 with respect to the examined groups of patients (Table 5).

### 3.5. Association between Serum Levels of IL-6, Zn, Alb, CRP, and Severity of Depressive Episode According to HAMD-17 and MADRS

The researched parameters were compared in each examined group considering the severity of the depressive episode on the Hamilton and Montgomery–Asberg Rating Scales.

Comparing depressive episodes of equal clinical severity (moderate or severe according to HAMD-17) in the studied patient groups (UD and BDD), there were no significant differences in serum levels of IL-6, Zn, CRP, and albumin.

In the groups of participants classified according to the diagnosis of affective disorder (UD or BD), with regard to the severity of the depressive episode assessed on the Hamilton Rating Scale for Depression, there were no significant differences in serum levels of IL-6, Zn, CRP, or Alb.

Interestingly, in patients with bipolar depression, severe depressive episode (evaluated according to Hamilton Scale), albumin values were significantly lower compared to the moderately severe depressive episode of the same participants group (Mann–Whitney U test, *p* = 0.042).

On this basis, there were no differences in the group with unipolar depression. Similarly, no significant differences were found when comparing moderate or severe depressive episodes assessed using the MADRAS between patients with bipolar and unipolar depression.

### 3.6. Correlations of Zn, Alb, CRP, and IL-6, with Items on the HAMD-17 and MADRS

Spearman’s correlation coefficient was used to evaluate the association of serum values of zinc, albumin, CRP, and IL-6 with individual items and the total score of HAMD-17 and MADRS.

We must underline the statistically significant positive and somewhat statistically weaker relationship between albumin and somatic anxiety (Rho = 0.264), and a negative and significant relationship with “hypochondriasis” (Rho = −0.303) in the group of subjects with unipolar depression (items on HAMD-17 scale). In addition, there is a significant and positive relation of CRP values to items “work and activities” (Rho = 0.253), “somatic symptoms, general” (Rho = 0.281), and item “weight loss” (Rho = 0.364). Interleukin-6 is significantly positively related to “work and activity” (Rho = 0.319), with “somatic symptoms, general” (Rho = 0.424) and with “weight loss” (Rho = 0.277), but negatively and significantly related to the scale item “guilt feelings” (Rho = −0.293). The total scale of HAM-D-17 in subjects with unipolar depression is not significantly related to the observed values (Table 6).

In groups of subjects with bipolar depression on the HAMD-17 scale, there was found a significant, positive, and slightly weaker relationship of zinc serum levels with scale items “gastrointestinal symptoms” (Rho = −0.366), “somatic symptoms, general” (Rho = −0.253), “weight loss” (Rho = −0.327), and also a positive and significant relationship with “anxiety-psychic” (Rho = 0.396). Albumin serum values were found to have a statistically significant and negative relation with “guilt feelings” (Rho = −0.341), and “work and activity” (Rho = −0.342). In addition, IL-6 serum values were found to be significantly statistically positively related to the items “gastrointestinal symptoms” (Rho = 0.254), “somatic symptoms, general” (Rho = 0.458), and with “work and activity” (Rho = 0.250), while a significant negative relationship with “anxiety-psychic” (Rho = −0.307) was calculated. The total HAMD-17 scale in subjects with bipolar depression is not significantly related to the observed values (Table 7).

Subjects with unipolar depression have a significant and positive relationship of CRP serum levels with the “concentration difficulties” item (Rho = 0.262), and IL-6 also with the items “concentration difficulties” (Rho = 0.278) and “lassitude” (Rho = 0.345) on the MADRS Depression Rating Scale (Table 8).

In subjects with bipolar depression, zinc values are significantly and negatively related to reduced appetite (Rho = −0.338). Albumin has a negative and significant relationship with noticeable grief (Rho = −0.259), reduced appetite (Rho = −0.264), concentration difficulties (Rho = 0.272), and the total MADRS (Rho = −0.253), and CRP has a positive and significant relationship related to fatigue (Rho = 0.384) (Table 9).

### 3.7. The Influence of Sociodemographic Data, General Medical Conditions and Substance Abuse Including Tobacco Smoking on the Severity of Depressive Episode According to HAMD and MADRS (Logistic Regression)

Bivariate and multivariate logistic regression analyses were conducted to predict the likelihood of a severe depressive episode using the Hamilton Depression Rating Scale (HAMD score of 25–52) and the Montgomery–Åsberg Depression Rating Scale (MADRS score of 35–60).

In the group of subjects with unipolar depression, a significant model in the bivariate regression for predicting a severe depressive episode was “pack years” (Odds Ratio [OR] = 1.15), while no significant model emerged in the multivariate regression. After correcting for the use of antidepressants, “pack years” remained a significant predictor in the unipolar depression group, indicating that patients with higher “pack years” had a 1.15 times greater chance of experiencing a severe depressive episode.

In the multivariate regression analysis, adjusted for antidepressant use, a significant model emerged that was entirely significant (χ^2^ test = 9.5, df = 1, *p* = 0.002) and explained between 35% (according to Cox & Snell) and 51% (according to Negelkerke) of the variance in severe depressive episodes, accurately classifying 77% of cases. In this model, “pack years” was a significant predictor (OR = 1.15).

In the group of subjects with bipolar depression, no significant predictors were found in the bivariate regression without antidepressant correction for predicting a severe depressive episode, nor did any significant model emerge in the multivariate regression. However, in the bivariate regression adjusted for antidepressant use, albumin levels were a significant predictor in the bipolar depression group, reducing the likelihood of a severe depressive episode (OR = 0.86).

In predicting the probability of a severe depressive episode, both with and without correction for antidepressant use, in the group of subjects with unipolar depression, no significant predictors or models were identified that would support the prediction of a severe depressive episode according to MADRS (total score 35–60).

## 4. Discussion

### 4.1. Comparison of Serum Levels of Zinc, Albumin, IL-6, and CRP between the Group of Depressive Subjects (UD and BDD) and Healthy Controls (HC)

In the first step, the difference in biological parameters between the group of patients and the group of healthy subjects was examined. The patient group displayed elevated levels of inflammatory markers IL-6 and CRP compared to healthy controls. This finding aligns with studies suggesting a key role for systemic inflammation in mood disorders, pointing towards immune dysregulation as a significant aspect of their pathophysiology and progression [13,14,36,37,38].

An interesting finding is the reduced level of zinc in the group of healthy controls.

This finding deviates from established patterns observed in previous studies, where individuals with depression typically exhibited lower serum zinc levels compared to healthy controls [26,39,40].

This is supported by research that investigated the therapeutic potential of zinc and consistently supported the effectiveness of zinc supplementation as an adjunctive treatment, showing improved mood in both depressed and healthy individuals [41,42,43,44]. Notably, zinc supplementation has also demonstrated benefits in improving mood among individuals with treatment-resistant depression [42].

Conventionally, depression is associated with reduced serum zinc levels, attributed to factors such as poor nutrition, increased physiological stress, and inflammation [45].

Possible explanations of these findings may be dietary variations that were not included in the study design, and which resulted in a lower zinc intake in the control group, influencing their serum levels. Additionally, there could be inherent physiological differences between the groups, particularly in how their bodies absorb and metabolize zinc, unrelated to the presence of depression. Another interesting angle to consider is the body’s response to stress in depression. It is conceivable that in depressed individuals, a unique stress response mechanism could paradoxically elevate zinc levels due to complex interaction between zinc, serotonin, and stress-related hormones in depression. These factors, individually or combined, might account for the unexpected findings and warrant further investigation to fully understand their implications [45].

Another finding was decreased albumin levels in the patient group (both UD and BD groups). This can be attributed to different influences including poor nutritional status, chronic inflammation, or acute phase response. Albumin, a key protein synthesized by the liver, is often reduced in chronic diseases and inflammatory states [46]. Its lower levels in the patient group could reflect the overall impact of mood disorders on physical health and metabolic function. The finding of significantly lower albumin levels in mood disorder [47] patients compared to healthy controls underscores the impact of these conditions on systemic health. Lower albumin levels in patients could indicate a state of chronic inflammation [46] or altered metabolism, a common occurrence in mood disorders [48].

### 4.2. Comparison of Serum Levels of Zinc, Albumin, IL-6, and CRP between the Groups of Unipolar Depressive Subjects (UD), Bipolar Depressive Subjects (BDD), and Healthy Controls (HC)

In the next step, the concentrations of zinc, albumin a, CRP, and IL-6 were compared between the groups of patients with unipolar depression (UD), bipolar depression (BDD), and the group of healthy subjects (HC).

This analysis found significantly lower serum levels of the inflammatory parameters IL-6 and CRP in the group of healthy subjects compared to both groups of patients (UD and BD), which is in accordance with our previous analysis, but also with existing the literature, suggesting the presence of an inflammatory component in mood disorders [49,50,51,52,53,54,55].

Elevated IL-6 and CRP levels have been associated with the pathophysiology of both unipolar and bipolar disorders, indicating systemic inflammation as a common characteristic in these conditions [14,56,57].

Perhaps the most striking finding is the lower zinc levels in the HC group compared to the UD group, diverging from the typical pattern where depressive states are often linked to reduced zinc due to poor nutrition or increased stress [26,45]. This suggests a complex interaction between zinc levels and mood disorders, potentially influenced by compensatory mechanisms in response to depression [58], dietary variations, or treatment effects [42,59].

Interestingly, zinc levels do not significantly differ between the BDD group and HC, suggesting unique aspects of zinc metabolism or homeostasis in BD [60]. This lack of difference could reflect specific physiological or compensatory responses in BD related to zinc regulation, distinct from those in UD [29]. Also, the impact of psychotropic medications on zinc levels might differ between unipolar and bipolar patients, partly due to the difference in medication regimens typically used in these conditions. Bipolar patients, who are often prescribed a combination of mood stabilizers, antipsychotics, and sometimes antidepressants, may experience more complex interactions affecting zinc levels compared to unipolar patients, who are more commonly treated with antidepressants alone. For instance, the fluctuating nature of bipolar disorder, with its manic and depressive episodes, may impact the immune system differently compared to the typically more persistent depressive state in unipolar depression [29].

### 4.3. Comparison of Serum Levels of Zinc, Albumin, IL-6, and CRP between the Groups of Unipolar Depressive Subjects (UD) and Bipolar Depressive Subjects (BDD)

A critical examination of inflammatory markers, nutritional elements, and protein levels has revealed a notable absence of significant differences between these two groups (UD and BDD) in parameters such as interleukin-6 (IL-6), C-reactive protein (CRP), zinc, and albumin.

This finding challenges the traditional dichotomy of unipolar and bipolar disorders, suggesting a more unified pathophysiological spectrum [61,62,63,64].

Both unipolar and bipolar disorders exhibit similar profiles in key inflammatory markers, IL-6 and CRP. This observation underscores a common inflammatory basis in mood disorders, as suggested by Bai et al. (2015) [14]. The elevated levels of these cytokines in both groups point toward a shared underlying inflammatory pathogenesis, potentially contributing to the symptomatology of both disorders. This convergence of inflammatory pathways in mood disorders echoes the growing recognition of immune dysregulation as a central aspect of psychiatric conditions [65,66,67,68].

Zinc levels, crucial for neurotransmitter function and neuroplasticity, have similarly shown no significant variance between unipolar and bipolar groups. The findings of Siwek et al. (2016) [29] lend credence to the hypothesis that alterations in zinc metabolism are a characteristic feature of depressive disorders, irrespective of their specific classification. This uniformity in zinc levels suggests that the role of zinc in mood regulation and brain function transcends the traditional diagnostic boundaries, hinting at a universal aspect of mood disorders.

Furthermore, the similarity in albumin levels across these groups might reflect a shared aspect of metabolic alteration in mood disorders. Albumin serum levels can be influenced by factors like nutrition, systemic inflammation, and overall health status [47,69]. The consistent levels across unipolar and bipolar disorders suggest parallel impacts on these physiological parameters.

The overarching implication of these findings is that unipolar and bipolar depression may share more common ground in their biological basis than previously acknowledged. This supports a more nuanced understanding of mood disorders as a continuum with shared biological mechanisms.

### 4.4. Comparison of the Results on Depression Rating Scales (HAMD-17 and MADRS) between the Examined Groups of Depressive Patients (UD and BD)

Both groups of patients were compared with respect to the scores on the Hamilton Scale and the MADRAS Scale for depression. The lack of significant differences in most HAMD-17 items between unipolar and bipolar depression groups suggests that the depressive episodes in these disorders manifest with similar symptom profiles. This finding aligns with previous research indicating overlapping features in the depressive phases of these mood disorders [9,32,70]. In clinical practice, UD and BDD can be difficult to diagnose.

The significant elevation of somatic anxiety in bipolar depression, compared to unipolar depression, as measured on the MADRS, could be attributed to the unique neurobiological and psychological aspects of bipolar disorder.

The heightened somatic anxiety in bipolar depression might be linked to the fluctuating nature of the disorder, where episodes of mania or hypomania precede or follow depressive phases, potentially leading to a heightened state of physiological arousal and anxiety [71,72]. Additionally, bipolar disorder may involve more pronounced dysregulation of stress response systems, which can manifest as somatic anxiety [73].

In conclusion, while unipolar and bipolar depression share many depressive symptoms, as shown in HAMD-17, the distinct increase in somatic anxiety in bipolar depression on the MADRAS highlights a key difference between these disorders. This underscores the importance of careful assessment in distinguishing between unipolar and bipolar depression, particularly considering the broader impact of bipolar disorder on somatic and psychological wellbeing.

### 4.5. Comparison of the Results of the Examined Groups of Depressive Patients (UD and BD) on the HAMD-17 and MADRS Rating Scales for Depression with Respect to the Severity of the Depressive Episode

The similarity in IL-6, Zn, CRP, and albumin levels between UD and BD during depressive episodes of similar severity suggests common underlying pathophysiological processes in mood disorders. This observation aligns with the growing evidence that mood disorders, irrespective of their specific type, may share fundamental inflammatory and metabolic disturbances [14].

The similar levels across UD and BD could indicate that these physiological aspects are consistently altered in depressive states. The shared biochemical profile in depressive episodes of UD and BD could be due to similar alterations in neuro-immune pathways, stress response mechanisms, or nutritional status associated with depressive states. It might also reflect the homogenizing effect of similar clinical severity on these biomarkers, regardless of the mood disorder subtype. This lack of distinction in key immuno-inflammatory markers between UD and BD depressive episodes challenges the long-held notion of these conditions as entirely separate entities. Instead, it proposes a continuum model of mood disorders where shared biological processes are involved [9,74]. Zinc, essential for neuroplasticity and immune function, further underscores the potential overlap in the neuro-immune pathways affected in both UD and BD [42,44]. However, the unique observation of lower albumin levels in severe episodes compared to moderate episodes within the BDD group may suggest that metabolic demands corelate with the severity of a depressive episode. Its reduction in severe BD episodes could reflect more pronounced systemic changes or stress responses, distinguishing severe BD from UD [58,75].

### 4.6. Correlations of the Examined Parameters (Zn, Albumin, IL-6, and CRP) in Both Groups of Depressive Subjects (UD and BD) with Items on the Depression Rating Scales (HAMD-17 and MADRS)

In the context of the research findings on unipolar and bipolar depression, a striking parallel emerges between the symptomatology of depression and so-called ‘sickness behaviour’. This comparison is particularly evident in the significant correlations found in unipolar depression between symptoms like somatic anxiety, hypochondriacal symptoms, general somatic symptoms, work and activity limitations, weight loss, concentration difficulties, and lassitude with levels of albumin, C-reactive protein (CRP), and interleukin-6 (IL-6) [76,77,78]. These correlations illuminate the critical role of inflammation (as indicated by CRP and IL-6 levels) and metabolic factors (as indicated by albumin levels) in the manifestation of depressive symptoms. Symptoms such as fatigue, reduced activity, physical discomforts, cognitive challenges, and negative emotional states are hallmark characteristics of ‘sickness behaviour’, underscoring a biological underpinning for these manifestations in depression.

In bipolar depression, the intricate relationship of zinc, albumin, IL-6, and CRP with the symptoms assessed using the HAMD-17 and MADRS scales reveals a more complex interaction. Lower zinc levels correlating with physical symptoms like gastrointestinal issues and weight loss, along with heightened psychic anxiety, are particularly noteworthy [79]. Moreover, the negative correlations of albumin with feelings of guilt, sadness, reduced appetite, and concentration difficulties, and the positive correlation of CRP with lassitude, closely mirror ‘sickness behaviour’ symptoms [76,77]. These findings suggest a significant contribution of both inflammatory processes and metabolic status to the breadth of depressive symptoms, resonating with the physical and psychological aspects of ‘sickness behaviour’.

The common thread running through both unipolar and bipolar depression is the overlap of symptoms such as lassitude, weight loss, gastrointestinal problems, reduced activity, and cognitive challenges with those typically associated with ‘sickness behaviour’. This overlap indicates that similar biological processes, including inflammation and metabolic alterations, might underpin these symptoms in both forms of depressive states and in ‘sickness behaviour’.

Understanding the nature of these correlations provides valuable insights into the mechanisms underlying depression and its resemblance to ‘sickness behaviour’. This knowledge lays a foundation for the exploration of targeted treatments that focus on these underlying biological factors. Recognizing the shared aspects of these conditions can lead to more effective management and treatment strategies, acknowledging the significant influence of biological markers on the symptomatology of depression. This perspective promotes a more holistic approach, addressing not just the psychological dimensions of depression, but also its biological aspects. This study is not without its limitations. First, the cross-sectional design of the research inhibits our ability to establish cause and effect relationships between the investigated biological parameters and depressive symptoms. Such designs can only provide a snapshot of data at a single point in time, which limits our understanding of how these relationships may evolve or respond to changes over time. Additionally, our study did not account for the onset of the disorders, nor the number of previous depressive episodes experienced by patients. This study design may have implications to the generalizability of our findings, as the trajectory and recurrence of depressive episodes can significantly influence biological markers and symptom presentation. These limitations should be carefully considered when interpreting the results, and should guide future research directions that might overcome these constraints.

## 5. Conclusions

The findings of our study confirm the presence of low-grade inflammation in depression. The results provide a better insight into the inflammatory hypothesis about the aetiology of depressive disorders. According to our findings, we cannot recommend zinc, albumin, interleukin-6, and CRP as specific biomarkers for distinguishing unipolar and bipolar depressive episodes.

## Figures and Tables

**Table 1:** General characteristics of the study population.

	Healthy Control (HC)	Patient Groups	Total	*p* *
Gender [n (%)]				
Female	60 (75)	84 (64.1)	144 (68.2)	0.10
Male	20 (25)	47 (35.9)	67 (31.8)	
Age (years) [Median (IQR)]	53 (44–57)	55 (47–58)	54 (42–57)	0.06 ^†^

* χ^2^ test; ^†^ Mann–Whitney U test.

**Table 2:** General characteristics of the study population distribution according to general characteristics and psychiatric heredity.

	Percentage of Participants (%)			*p* *
	Unipolar Depression	Bipolar Depression	Total	
Educational Level				
No formal education	1 (2)	1 (2)	2 (2)	0.004
Elementary school	10 (15)	9 (14)	19 (15)	
High school	44 (67)	31 (47)	75 (56)	
College (short program)	9 (14)	9 (13)	18 (14)	
University degree	1 (2)	16 (24)	17 (13)	
Employment Status				
Employed	27 (42)	32 (48)	59 (45)	0.633
Unemployed	18 (28)	14 (21)	32 (24)	
Retired	20 (30)	20 (31)	40 (31)	
Marital Status				
Married	41 (64)	37 (55)	78 (60)	0.418 ^†^
Divorced	9 (15)	13 (20)	22 (17)	
Single	10 (15)	14 (22)	24 (18)	
Widowed	5 (7.6)	2 (3)	7 (5)	
Psychiatric Heredity	18 (28)	24 (36)	42 (32)	0.288
Family History of Psychiatric Illness (n = 40)				
Mother—depressive disorder	6 (33.3)	12 (54.5)	18 (45)	0.498 ^†^
Father—depression	1 (5.6)	1 (4.5)	2 (5)	
Father—alcoholism, suicide	1 (5.6)	0	1 (3)	
Father—alcoholism	4 (22)	5 (22.7)	9 (23)	
Mother—bipolar disorder	0	2 (9.1)	2 (5)	
Sister—depression	1 (5.6)	1 (4.5)	2 (5)	
Sister—schizophrenia	1 (5.6)	0	1 (3)	
Father—suicide	1 (5.6)	0	1 (3)	
Sister and mother suicide, father alcoholic	1 (5.6)	0	1 (3)	
Brother PTSD, brother alcoholism, mother BAP	0	1 (4.5)	1 (3)	
Father and brother depression and suicide	1 (5.6)	0	1 (3)	
Aunt depressive disorder	1 (5.6)	0	1 (3)	

* χ^2^ test; ^†^ Fisher’s exact test.

**Table 3:** Comparison of serum levels of IL-6, CRP-u, albumin, and zinc between the healthy control group and patient group.

	Median (Interquartile Range)		Difference	95% CI (Confidence Interval)	*p* *
	Healthy Controls	Patients			
IL-6	0.95 (0.296–2.518)	1.54 (0.871–2.35)	0.41	0.14–0.76	0.006
CRP	1.40 (0.90–2.15)	2.90 (1.10–6.0)	0.80	0.10–2.20	0.002
Albumin	46.0 (44.0–48.0)	44.05 (42.0–47.0)	−2	−3–(−1)	0.002
Zinc	12.20 (10.67–14.02)	13.20 (11.43–15.38)	1.22	0.23–2.11	0.013

* Mann–Whitney U test.

**Table 4:** Differences between IL-6, CRP, albumin, and zinc between healthy the control group and patient group, considering the spectrum of the disorder (unipolar; bipolar).

	Median (Interquartile Range)			*p* *
	Control Group	Unipolar Depression	Bipolar Depression	
IL-6	0.95 (0.296–2.518)	1.54 (0.82–2.76)	1.53 (0.96–2.26)	0.020 ^†^
CRP	1.40 (0.90–2.15)	3.40 (1.0–5.70)	2.45 (1.10–6.80)	0.008 ^†^
Albumin	46.0 (44.0–48.0)	44.80 (42.10–47.0)	44.0 (42.0–47.0)	0.007 ^†^
Zinc	12.20 (10.67–14.02)	13.40 (11.9–15.5)	13.15 (10.40–15.0)	0.013 ^‡^

* Kruskal Wallis test (post hoc Conover); ^†^ *p* < 0.05 significantly lower values in controls vs. unipolar and bipolar spectrum; ^‡^ *p* < 0.05 significantly lower values in controls vs. unipolar spectrum.

**Table 5:** Comparison of serum levels of zinc, albumin, CRP, and IL-6 between groups of participants with unipolar and bipolar depression.

	Median (Interquartile Range)		Difference	95% CI (Confidence Interval)	*p* *
	Unipolar Depression	Bipolar Depression			
Zinc	13.4 (11.9–15.5)	13.2 (10.4–15)	−0.8	−1.7–0.3	0.134
Albumin	44.8 (42.1–47.0)	44.0 (42–47)	0	−1.6–1.0	0.825
CRP	3.4 (1.0–5.7)	2.5 (1.1–6.8)	0.1	−0.7–0.8	0.718
IL-6	1.54 (0.82–2.76)	1.53 (0.96–2.26)	−0.006	−0.44–0.36	0.962

* Mann–Whitney U test.

**Table 6:** Association between items on HAMD-17 in groups of patients with unipolar depression (Spearman’s correlation coefficient).

Unipolar Depression Items on HAMD-17 Scale	Spearman’s Correlation Coefficient Rho (*p* Value)			
	Zinc	Albumin	CRP	IL-6
Depressed mood	0.085 (0.50)	−0.087 (0.49)	0.019 (0.88)	−0.035 (0.79)
Guilt feelings	0.019 (0.88)	−0.103 (0.42)	0.006 (0.96)	−0.293 (0.02)
Suicide thoughts	0.040 (0.75)	−0.160 (0.21)	0.037 (0.77)	0.074 (0.56)
Insomnia-early	0.093 (0.46)	0.060 (0.64)	0.020 (0.88)	0.093 (0.47)
Insomnia-middle	−0.031 (0.80)	0.049 (0.70)	−0.024 (0.85)	0.002 (0.99)
Insomnia-late	0.130 (0.30)	0.160 (0.21)	0.014 (0.91)	−0.112 (0.38)
Work and activities	−0.226 (0.07)	−0.238 (0.06)	0.253 (0.04)	0.319 (0.01)
Retardation psychomotor	0.008 (0.95)	−0.193 (0.13)	0.166 (0.19)	0.221 (0.08)
Agitation	0.238 (0.06)	0.077 (0.55)	−0.115 (0.36)	−0.063 (0.62)
Anxiety-psychic	−0.035 (0.78)	0.079 (0.54)	0.249 (0.05)	0.114 (0.37)
Anxiety-somatic	0.217 (0.08)	0.264 (0.04)	0.011 (0.93)	−0.054 (0.67)
Gastrointestinal symptoms	−0.247 (0.05)	−0.104 (0.41)	0.064 (0.61)	0.091 (0.48)
Somatic symptoms. general	−0.227 (0.07)	−0.159 (0.21)	0.281 (0.02)	0.424 (<0.001)
Sexual disturbances	−0.082 (0.52)	−0.016 (0.90)	0.083 (0.51)	0.107 (0.41)
Hypochondriasis (somatization)	−0.109 (0.39)	−0.303 (0.01)	0.095 (0.45)	0.122 (0.34)
Weight loss	−0.231 (0.06)	−0.249 (0.05)	0.364 (<0.001)	0.277 (0.03)
Insight	−0.074 (0.56)	−0.164 (0.19)	0.046 (0.72)	0.166 (0.19)
HAMD−17 total	−0.027 (0.83)	−0.091 (0.47)	0.227 (0.07)	0.177 (0.16)

**Table 7:** Association of serum levels of Zn, Albumin, CRP, and IL-6 to HAMD-17 items in a group of patients with bipolar depression.

Bipolar Depression	Spearman’s Correlation Coefficient Rho (*p* Value)			
	Zinc	Albumin	CRP	IL-6
Depressed mood	−0.072 (0.57)	−0.239 (0.05)	0.052 (0.68)	0.133 (0.29)
Guilt feelings	−0.110 (0.38)	−0.341 (0.01)	0.145 (0.25)	0.050 (0.69)
Suicidal thoughts	−0.001 (0.99)	−0.174 (0.16)	−0.071 (0.57)	0.037 (0.77)
Insomnia: initial	0.050 (0.69)	−0.069 (0.58)	0.154 (0.22)	−0.053 (0.68)
Insomnia: middle	0.082 (0.51)	0.124 (0.32)	0.194 (0.12)	−0.104 (0.41)
Insomnia: late	0.164 (0.19)	0.205 (0.1)	0.187 (0.13)	−0.084 (0.51)
Work and activities	−0.182 (0.14)	−0.342 (<0.001)	0.093 (0.46)	0.250 (0.04)
Psychomotor retardation	0.125 (0.32)	0.043 (0.73)	−0.008 (0.95)	0.039 (0.76)
Psychomotor agitation	0.176 (0.16)	−0.062 (0.62)	0.019 (0.88)	−0.225 (0.07)
Anxiety. psychic	0.396 (<0.001)	0.059 (0.64)	0.040 (0.75)	−0.307 (0.01)
Anxiety. somatic	0.088 (0.48)	0.082 (0.51)	0.202 (0.10)	−0.150 (0.23)
Gastrointestinal symptoms	−0.366 (<0.001)	−0.231 (0.06)	0.225 (0.07)	0.254 (0.04)
Somatic symptoms. general	−0.253 (0.04)	−0.270 (0.03)	0.206 (0.10)	0.458 (<0.001)
Sexual disturbances	0.035 (0.78)	0.075 (0.55)	0.046 (0.71)	−0.009 (0.94)
Hypochondriasis (somatization)	−0.111 (0.38)	−0.011 (0.93)	0.206 (0.10)	0.032 (0.80)
Weight loss	−0.327 (0.01)	−0.129 (0.30)	0.122 (0.33)	0.152 (0.23)
Insight	−0.018 (0.89)	−0.095 (0.45)	0.028 (0.82)	−0.105 (0.40)
HAMD-17 total	−0.082 (0.51)	−0.211 (0.09)	0.239 (0.05)	0.024 (0.85)

**Table 8:** Association of items on MADRS to serum levels of Zn, albumin, CRP, and IL-6 in groups of patients with unipolar depression.

Unipolar Depression	Spearman’s Correlation Coefficient Rho (*p* Value)			
	Zinc	Albumin	CRP	IL-6
Apparent sadness	0.079 (0.53)	0.068 (0.59)	0.238 (0.06)	0.113 (0.38)
Reported sadness	0.171 (0.17)	0.003 (0.98)	0.087 (0.49)	−0.059 (0.65)
Inner tension	−0.019 (0.88)	−0.200 (0.11)	0.138 (0.27)	0.160 (0.21)
Reduced sleep	0.135 (0.28)	−0.045 (0.73)	0.165 (0.19)	0.056 (0.67)
Reduced appetite	0.006 (0.96)	−0.167 (0.19)	−0.065 (0.61)	0.081 (0.53)
Concentration difficulties	−0.170 (0.18)	−0.181 (0.15)	0.262 (0.04)	0.278 (0.03)
Lassitude	−0.191 (0.13)	−0.051 (0.69)	0.228 (0.07)	0.345 (0.01)
Inability to feel	−0.115 (0.36)	−0.029 (0.82)	0.114 (0.37)	0.109 (0.39)
Pessimistic thoughts	−0.049 (0.70)	−0.163 (0.20)	0.200 (0.11)	0.070 (0.59)
Suicidal thoughts	−0.109 (0.39)	−0.053 (0.68)	0.221 (0.08)	0.238 (0.06)
MADRS total	−0.005 (0.97)	−0.124 (0.33)	0.219 (0.08)	0.184 (0.15)

**Table 9:** Association of items on MADRS to serum levels of Zn, albumin, CRP, and IL-6 in groups of patients with bipolar depression.

Bipolar Depression	Spearman’s Correlation Coefficient Rho (*p* Value)			
	Zinc	Albumin	CRP	IL-6
Apparent sadness	0.029 (0.82)	−0.259 (0.04)	0.120 (0.34)	0.062 (0.62)
Reported sadness	−0.055 (0.66)	−0.201 (0.11)	0.167 (0.18)	0.070 (0.58)
Inner tension	0.002 (0.99)	−0.062 (0.62)	0.099 (0.43)	−0.153 (0.22)
Reduced sleep	0.096 (0.44)	−0.130 (0.30)	0.118 (0.34)	−0.012 (0.92)
Reduced appetite	−0.338 (0.01)	−0.264 (0.03)	0.159 (0.20)	0.151 (0.23)
Concentration difficulties	−0.051 (0.69)	−0.272 (0.03)	0.135 (0.28)	0.101 (0.42)
Lassitude	−0.174 (0.16)	−0.231 (0.06)	0.384 (<0.001)	0.245 (0.05)
Inability to feel	−0.165 (0.19)	−0.221 (0.08)	0.131 (0.29)	0.073 (0.57)
Pessimistic thoughts	−0.068 (0.59)	−0.183 (0.14)	0.136 (0.28)	−0.046 (0.72)
Suicidal thoughts	−0.156 (0.21)	−0.172 (0.17)	0.075 (0.55)	0.102 (0.42)
MADRS total	−0.066 (0.60)	−0.253 (0.04)	0.185 (0.14)	0.064 (0.61)

## Data Availability

Data is unavailable due to privacy or ethical restrictions.
